# Acute and Subacute Toxicity of Methanol Extract of* Syzygium guineense* Leaves on the Histology of the Liver and Kidney and Biochemical Compositions of Blood in Rats

**DOI:** 10.1155/2019/5702159

**Published:** 2019-03-10

**Authors:** Million Loha, Abay Mulu, Solomon M. Abay, Wondwossen Ergete, Bekesho Geleta

**Affiliations:** ^1^Department of Anatomy, School of Medicine, College of Health Sciences, Wachemo University, Hosaena, Ethiopia; ^2^Department of Anatomy, School of Medicine, College of Health Sciences, Addis Ababa University, Addis Ababa, Ethiopia; ^3^Department of Pharmacology, School of Medicine, College of Health Sciences, Addis Ababa University, Addis Ababa, Ethiopia; ^4^Department of Pathology, School of Medicine, College of Health Sciences, Addis Ababa University, Addis Ababa, Ethiopia; ^5^Directorate of Traditional and Modern Medicine Research, Ethiopian Public Health Institute, Addis Ababa, Ethiopia

## Abstract

Plant medicine is the oldest form of health care known to mankind.* Syzygium guineense* is one of the many species of Ethiopian medicinal plants which has a long history of use as remedies for various ailments such as dysentery, diarrhea, and hypertension. In many countries, herbal medicines and related products are introduced into the market without safety or toxicological evaluation. The aim of this study was to investigate the histopathological effect of 80% methanol extract of* S. guineense* on liver and kidney and blood parameters of rats. For acute toxicity study, rats were randomly divided into three groups (n=4). The control group received distilled water, while the experimental groups received a single dose of 2000 mg/kg and 5000 mg/kg 80% methanolic extract of* S. guineense *leaves per oral. For subacute toxicity study, the rats were randomly divided into three groups (n=6). The control group received distilled water, while the experimental groups received 500 mg/kg and 1500 mg/kg 80% methanol extract of* S. guineense* leaves orally for 28 days. At the end of the experiment, blood samples were collected for hematology and clinical chemistry evaluations. Gross pathology and histopathology of liver and kidneys were assessed. In the acute toxicity study, rats treated with 2000 mg/kg and 5000 mg/kg showed no toxicological signs observed on behavior, gross pathology, and body weight of rats. In the subacute toxicity study rats have showed no significant changes on behavior, gross pathology, body weight, and hematological and biochemical parameters, whereas both experimental groups had a lower blood glucose level compared with the control group (p < 0.05). There were no significant differences in the gross and histopathology of the liver and kidneys of experimental animals in extract exposed groups and their counterpart control. The 80% methanol extract of* S. guineense* does not produce adverse effects in rats after acute and subacute treatment. Before marketing a* S. guineense* leaf based remedy, subchronic and chronic toxicity evaluations need to be done.

## 1. Background

Plant medicine is the oldest form of health care known to mankind. Herbal medicine flourishes today as the primary form of medicine for perhaps as much as 80% of the world's population [1]. Usually, a specific part of the plant (root, leaves, fruit, flowers, and seeds) is used in traditional preparations or as pure active principles formulated into a suitable preparation. Many medicines commonly used today are of herbal origin. Indeed, about 25% of prescription drugs contain at least one active ingredient derived from plant material [2, 3].

Plant derived medicines are used in all civilizations and cultures and, hence, plants have always played a key role in health care systems worldwide. In most developing countries, the indigenous modes of herbal treatment are parts of the culture and the dominant method of healing therapy. These remedies, with a considerable extent of effectiveness, are socially accepted and economically viable and, mostly, are the only available source [4, 5].

The various literature available shows the significant role of medicinal plant in primary health care delivery in Ethiopia where 70% of human and 90% of livestock population depend on traditional medicine like many developing countries particularly that of sub-Saharan African countries [6]. The Ethiopian Flora is estimated to consist of between 6000 and 7000 species distributed in about 245 plant families. Although the exact number is still unknown, many the species, i.e., about one-third of the families, have been employed in traditional medicinal practices [7].

Almost any substance can be harmful at some doses but, at the same time, can be without harmful effect at some lower dose. Between these two limits there is a range of possible effects, from subtle long-term chronic toxicity to immediate lethality [8]. The large array of toxic chemicals produced by plants (phytotoxins), usually referred to as secondary plant compounds, is often held to have evolved as defense mechanisms against herbivorous animals, particularly insects and mammals. Many chemicals that have been shown to be toxic are constituents of plants that form part of the human diet [8].

*Syzygium guineense *(Willd) D.C (*Myrtaceae*), Vernecular name "Dokima” (Amharic), in English as “water berry” and in Afaan Oromoo “Baddessaa”, is a medium sized or tall ever green tree 15-30m high with edible fruits. It is widely distributed in sub-Saharan Africa [9, 10].* S. guineense* is an odorous species native of the wooded savannahs and tropical forests of Africa [11]. Traditionally, many morphological parts of this plant is used for management of various ailments such as malaria [12], its fruits and bark for dysentery and diarrhea, infusion prepared from its leaves, fruits, or bark for management of hypertension [13].

The studies also indicated that the methanol extract of the bark has a hypotensive effect [14], antihypertensive and vasodilatory effect [15], analgesic and anti-inflammatory effect [16], antimalarial activity [17], and antidiabetic activity [18]. Its bark is used in traditional medicine to treat gastointestinal upsets and diarrhea [19–21]. The twigs and leaves have anthelmintic (hookworm). Its watery extract of fruits and bark has activity against different strains of bacteria responsible for diarrhea [22, 23]. Another study conducted in Cameroon showed that its leaf has antioxidant properties and beneficial activities on oxidative stress [24].

The chemical composition of* S. guineense* leaves includes pectic polysaccharides and two immunologically active polysaccharide fractions such as arabinogalactan type II polysaccharide, called Sg50A1; another polysaccharide fraction is a mixture of oligosaccharides of the pectic type, called Sg50A2 [25]. Its wild, oval fruits are edible with high concentrations of Ca, Mg, Fe, K, and P [26, 27]. Antibacterial activity of triterpenes isolated from fruits and bark of* S. guineense* has been demonstrated [28]. Phytochemical screening of the plant revealed that* S. guineense* leaf extract contains flavonoids, polyphenols, tannins, saponins, carbohydrate, alkaloids, cardiac glycosides, proteins, lipids, polyphenols, steroids, and coumarins. These phytochemical constituents are physiologically active compounds possessing great potential for therapeutic and prophylactic uses [16, 24].

Despite these all efficacy studies on various parts of the* S. guineense*, there is a limited safety study conducted for this medicinal plant. Hence it has become necessary to standardize the safety and quality assurance measures to ensure supply of medicinal plant materials of good quality [5]. One of the important components of this study was scientific evaluation of safety and toxicity of* S. guineense.* The outcome of this study may fill the gap of the previous studies on* S. guineense *and also it is hoped to provide some additional evidence for recommending further studies to assess toxicity profiles associated with the use of herbal preparations of this plant. Taking into consideration the rationale, the objective of the study is to investigate the acute and subacute histopathological of leaves of 80% methanol extract of* S. guineense* on liver and kidney and blood parameters of rats.

## 2. Materials and Methods

### 2.1. Collection and Extraction of Plant Materials

The leaves of* S. guineense *were collected from Wondogenet, around Shashemene town, located about 270 km south of Addis Ababa in September, 2015. Then the leaves were identified and authenticated by a taxonomist at Ethiopian Public Health Institute (EPHI) and a voucher number AL-002 was deposited in the herbarium for future reference. The plant materials were cleaned of extraneous materials and 80% methanol extract of* S. guineense *was prepared for the study. The powdered leaves were macerated with 80% methanol in water for 72 hrs with intermittent agitation by orbital shaker DS-500. Then, the supernatant part of agitated materials was separated from the undissolved portion of the plant material. The supernatant portion was filtered with 0.1 mm^2^ mesh gauze and then with Whatman grade 1 filter paper with pore size of 11*μ*m. The filtrate was then concentrated by evaporating the solvent using a rotary evaporator (BUCHI Rota-vapor type R-205, Switzerland) under reduced pressure at a temperature of 40–45°C. Then the residue was dried by steam bath at 40°C for period of one week to make it dry [29].

### 2.2. Preparation and Grouping of Experimental Animals

The healthy and nonpregnant Wistar rats of both sex with age of 8 to 10 weeks and above and weight of 120g–140 g were obtained from Physiology Department, Addis Ababa University. The animals were acclimated to laboratory conditions for 5 days. They were housed in standard cages and kept under standard condition at a temperature of 22°C (± 3°C), with 12hrs light/12hrs dark cycle [30]. They were provided with free access to standard diet and tap water* ad libitum* [31].

Animals were randomly assigned to a control and two treatment groups. Each animal was assigned a unique identification number. A total of 18 rats of both sex containing 6 rats per group (three female and three male) were used for subacute toxicity study, whereas 12 female rats containing four female rats per group were used for acute toxicity study: a control and two treatment groups [30].

### 2.3. Acute Toxicity Study

According to OECD guideline [30] normal females, nulliparous and nonpregnant were used. Before conducting the experiment, the animals were randomly selected and grouped into three group (n=4) and then kept in their cage for 5 days prior to dosing to allow acclimatization to the laboratory conditions. All groups of the rats fasted overnight prior to administration. Following the fasting period, all animals were weighed, and the doses were calculated based on their body weight. The extracts were prepared in distilled water.

The 80% methanol extract of the leaves was then administered orally at the doses of 2000 mg/kg (group I) and 5000 mg/kg (group II) body weight of rats in the test groups. Control group (group III) received 80% methanol. These doses were selected based on the previous efficacy studies. After administering the plant materials, the animals were kept under close observation continuously for 1 hour and intermittently for 4 hours and thereafter once every 24 hours for the next 14 days.

During this study period, clinical observations were made for mortality, behavioral, neurological, and any other abnormalities and their weight was measured weekly. Finally, on the 15^th^ day, their final weights were measured, and gross physical examinations were carried out. The rats were then anesthetized under diethyl ether. After sacrificing the rats, gross pathological observation was carried out on vital organs.

### 2.4. Subacute Toxicity Study

The subacute toxicity study was conducted for 28 days to examine the toxicity of the extract on some blood parameters and histopathology of the liver and kidneys [30]. For this study healthy adult rats of both sexes were used. Eighteen rats were randomly distributed into three groups (I, II, and III) each consisting of six rats (three female and three male) per group. Groups I and II were orally administered with 80% methanol extract of leaves at doses of 500 and 1500 mg/kg body weight per day, respectively, for 28 days using oral gavage. Group III served as control group and received 80% methanol, the vehicle to dissolve the extract. Clinical observation was carried out for 28 days and their weight was measured weekly for four weeks. On the 28^th^ day the final weight of the rats was measured and then they were anesthetized under diethyl ether and blood samples were collected from each animal by cardiac puncture.

The blood was placed in two groups of test tubes, half of the test tubes containing anticoagulant, ethylene diaminotetraacetic acid (EDTA), and the other half without anticoagulant. Blood samples in the test tubes containing EDTA were used to determine the hematological parameters (WBCs, RBCs, HGB, HCT, MCH, MCHC, and platelets) using Automated Hematology Analyzer (Symex-RX, 21, Japan). Blood samples in the test tubes without anticoagulant could clot and sera were obtained by centrifuging the blood using an electrical centrifuge (HUMAX-K, HUMAN-Germany) from which blood chemistry (glucose, urea, creatinine, total protein, ALT, and AST) was studied to test renal and hepatic functions. Values in the sera were analyzed using Automated Clinical Chemistry Analyzer (AUTO LAB 18, clinical chemistry analyzer, Italy). After collection of blood samples, the rats were sacrificed by cervical dislocation and parts of the liver and the kidney were dissected out; and gross pathological observation was performed on liver and kidney to check for any gross lesions.

### 2.5. Histopathological Studies

The liver and kidney sections taken randomly for tissue processing were fixed in 10% neutral buffered formalin (NBF) overnight at room temperature. After fixation, the tissue sections were washed with water to remove excess fixatives for about six hours and dehydrated with increased concentration of alcohol of 70% for two hours, 90% for two hours, absolute alcohol-I, II for one and half hours, and III overnight. The dehydrated tissues were cleared in two changes of xylene-for one and half hours and two and half hours. The tissues were then infiltrated with three changes of paraffin wax-for one and half hours, two and half hours, and overnight. Finally, the tissues were embedded in paraffin wax in square metal plates forming tissue blocks, whereby each tissue block was labeled and stored at room temperature till sectioned.

The tissue blocks were sectioned in ribbons at a thickness of 5 *μ*m with Leica microtome (Leica RM 2125RT Nussloch GmbH, Germany). The ribbons of the section were collected at every 5^th^ sections and put onto the surface of a warm water bath of temperature of 40°c. The floating ribbons over the surface of warm water were mounted onto precleaned slides spread with egg albumin. The slides containing paraffin wax were arranged within the slide holder and placed in an oven with temperature of 40°c for about 20 minutes so as to fix the tissue to the slides and allowed to cool at room temperature for 30 minutes and stained regressively with routine Harris haematoxylin for 6 minutes and then eosin for 17-20 second (H and E).

For routine H and E staining, two series of coupling jars were prepared: one for paraffin removal and hydration and the other for dehydration and clearing. So, sections were placed in xylene I for 5 minutes and xylene II for 2 minutes again to remove the paraffin from tissue and hydrated with decreasing concentrations of absolute I, II and 95% alcohol for two minutes each, 70% of alcohol for three minutes, and 50% alcohol for five minutes. The tissue sections were washed with tap water for five minutes and stained regressively with Harris haematoxylin for 6 minutes and then washed under running tape water for five minutes again. The slides were immersed in acidic alcohol for differentiation and controlling over stained haematoxylin for 1 second and then put in bluing solution (sodium bicarbonate) until they became blue. After bluing, the slides were counter stained with eosin for 17-20 seconds and then washed in tap water for two minutes. The sections were dehydrated with increasing alcohol concentration of 50%, 70%, 95%, absolute I and II for two minutes each. The dehydrated sections were cleared with xylene I and xylene II for three minutes each and permanently mounted on microscopic slides using DPX and cover slips and then observed under light microscope for the investigations of any histological change, thereby the histology of the treated groups was compared with histology of the control group. After examination, photomicrographs of selected samples of liver and kidney section from both the treated and control rats were taken under a magnification of x20 objective using (EVOS XL, USA) automated built-in digital photo camera.

### 2.6. Statistical Analysis

Data were presented as mean ± SEM with 95% confidence interval and analyzed by SPSS version 22. And one way ANOVA followed by* post hoc *test (Dunnett's T3) was used for multiple comparisons of the mean differences and responses of different doses of extracts. The difference between groups with respect to variables under investigation was significant at P value of less than 0.05.

### 2.7. Ethical Consideration

The study was conducted after having approval from the Ethical and Scientific committee of the Department of Anatomy, Addis Ababa University, and in line with the highest standard for the humane and compassionate use of animals in biomedical research. Animals used in this study were not subjected to any unnecessary painful and terrifying situations [30]. To keep the pain and suffering minimal during any surgical intervention all animals were given diethyl ether anesthetic and the procedure was carried out by a well-trained person. The animals were protected from pathogens and placed in appropriate environment. The numbers of animals were reduced to the minimum possible that allows investigators achieving the scientific objectives of the study [31].

## 3. Results

### 3.1. Acute Toxicity Study

The acute toxicity study did not show any toxicity sign and symptom at 2000mg/kg and 5000mg/kg. No morbidity or mortality was observed in the treated groups at both doses during acute toxicity study. As a result, the LD_50_ of the extract could be greater than 5000mg/kg body weight.

The gross pathological studies on the liver and kidneys of treated rats showed no significant abnormal changes in color, size, shape, and texture compared with the control. The mean absolute weights of the liver were 5.98 ± 0.82 g (at 2000mg/kg), 5.03 ± 0.55g (at 5000mg/kg) compared with the control (5.40 ± 1.81) g. The mean absolute weights of the kidneys were 1.10 ± 0.18 g (at 2000mg/kg) and 1.15 ± 0.16 g (at 5000mg/kg) compared with the control (1.15 ± 0.54) g (Table 1).

There was a gradual increase in the body weight of both the treated and control rats (Table 2). The initial mean body of control rats was 127.8250 ± 5.62604 g; at the end of the experiment their final mean body weight was 146.9250 ± 9.30245 g. The mean body weight gain for the control rats was 19.10 g.

The initial mean body weights of rats treated with doses of 2000mg/kg, and 5000mg/kg were 126.8250 ± 3.13645 g, and 125.3250 ± 3.03682 g respectively. At the end of the experiment (after 15 days) the final mean body weight of rats treated with 2000mg/kg and 5000mg/kg was 145.7250 ± 5.48610 g, and 143.1000 ± 6.80012 g, respectively. The mean body weight gain for rats treated with 2000mg/kg, and 5000mg/kg was 18.9 g and 17.8 g, respectively.

### 3.2. Subacute Toxicity Study

#### 3.2.1. Effects on the Behavior, Gross Pathology, Organ, and Body Weight

**Figure 1:**
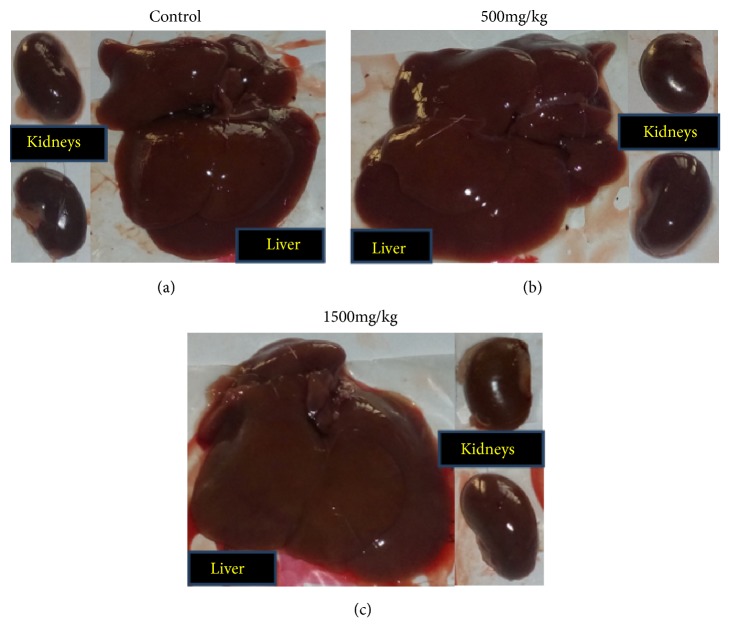
Photograph of liver and kidneys from the subacute toxicity study.

Throughout the study period no sign of toxicity and mortality was observed on treated rats, which received 500 mg/kg and 1500 mg/kg. Gross observation of the liver and kidneys of the treated rats showed no significant changes compared with the control group (Figure 1); and no significant difference (P > 0.05) was observed in the mean absolute organs weight between control and treated groups. The mean absolute weights of the liver were 7.70 ± 1.07 g (at 500mg/kg) and 6.40 ± 0.97 g (at 1500mg/kg), compared with the control (7.03 ± 1.02 g). Similarly, the mean absolute weights of the kidneys of rats were in the control and extract treated groups was not significantly different (Table 3).

**Figure 2:**
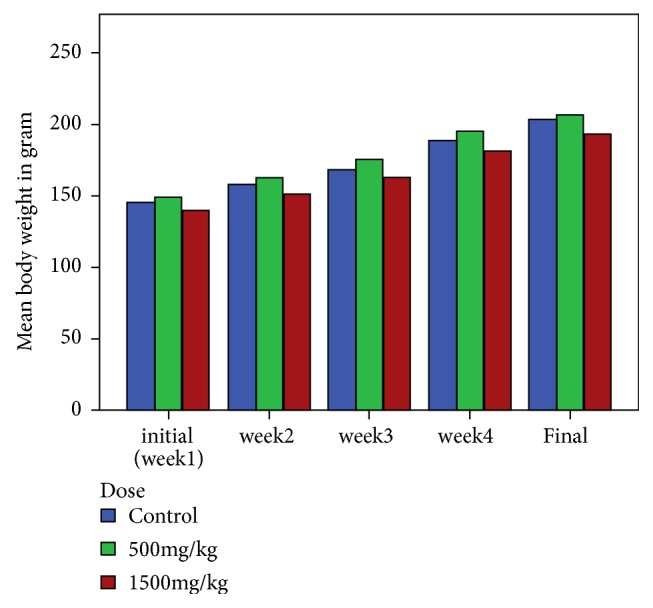
Bar graph of mean body weight change in rats treated with 500mg/kg and 1500mg/kg of 80% methanol extract as compared to the control group during subacute toxicity study.

During the subacute experimental period all groups of rats showed gradual and normal increase in their body weight (Figure 2). However, there was no statistically significant (p > 0.05) weight difference between the treated and control groups. The initial mean body weight of control group was 144.70* ± *21.89 g, and final mean body weight was 203.22* ± *31.07 g. The initial mean body weight of rats treated with the dose of 500 mg/kg was 148.48* ± *11.94 g, and final mean body weight was 206.90* ±* 17.19 g. The initial mean body weight of rats treated with the dose of 1500 mg/kg was 139.80* ± *9.26 g, and the final mean body weight was 193.48* ±* 16.63 g.

#### 3.2.2. Effects on Hematological Parameters

In the subacute toxicity study, the hematological parameters, such as WBC, RBC, PLT, HGB, HCT, MCV, MCH, and MCHC, of the treated groups (500 mg/kg and 1500 mg/kg) were within the reference range for rats. The values in the extract treated rats were not significantly different from the control (Table 4).

#### 3.2.3. Effects on Biochemical Parameters

In the subacute toxicity study, the biochemical parameters (except glucose) of the treated groups (500 mg/kg and 1500 mg/kg) were within the reference range for rats and were not significantly different from the control group. The level of urea in both treated grouped increased even though not statistically significant (p > 0.05). The mean values of serum glucose were 53.83* ± *4.11 mg/dl at 500 mg/kg and 61.83* ± *10.77 mg/dl at 1500 mg/kg, while 80.83* ±* 6.62 mg/dl for the controls. The mean serum glucose level showed significant decrease (p < 0.05) at both doses compared with the controls. However, the change in serum glucose between two doses (500 and 1500mg/kg) was not significant (p = 0.294) (Table 5)

#### 3.2.4. Effects on Histology of the Liver

**Figure 3:**
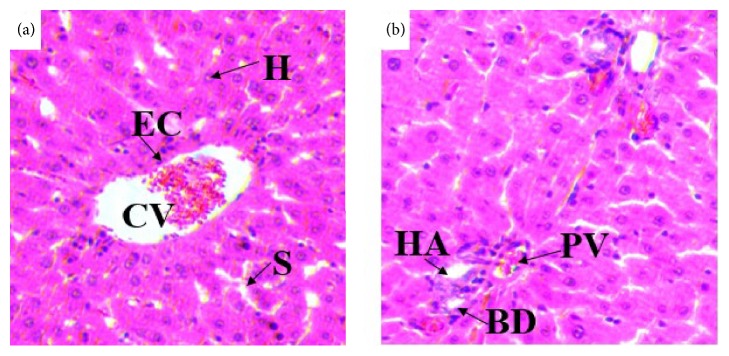
(a) and (b) Photomicrographs of liver section of control rats (H and E, X400). CV= central vein, EC= endothelial cells, H= hepatocytes, KC= Kupffer cells, BD= bile duct, HA= hepatic artery, and PV= portal vein.

**Figure 4:**
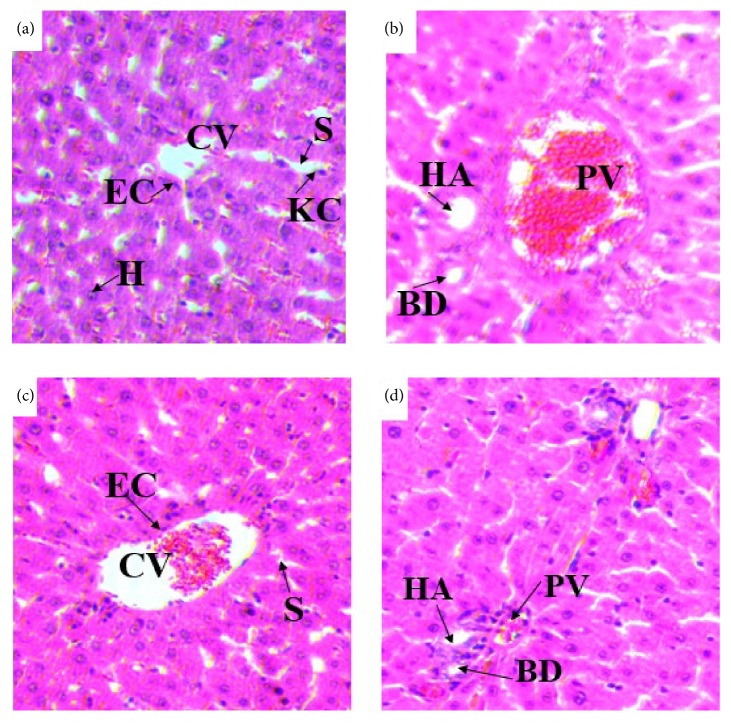
(a) and (b) Photomicrographs of liver section of rats treated with 500mg/kg of 80% methanol extract of* S. guineense*. (c) and (d) Liver section of control rats (H and E, X400). CV= central vein, EC= endothelial cells, H= hepatocytes, KC= Kupffer cells, S= sinusoids, BD= bile duct, HA= hepatic artery, and PV= portal vein.

**Figure 5:**
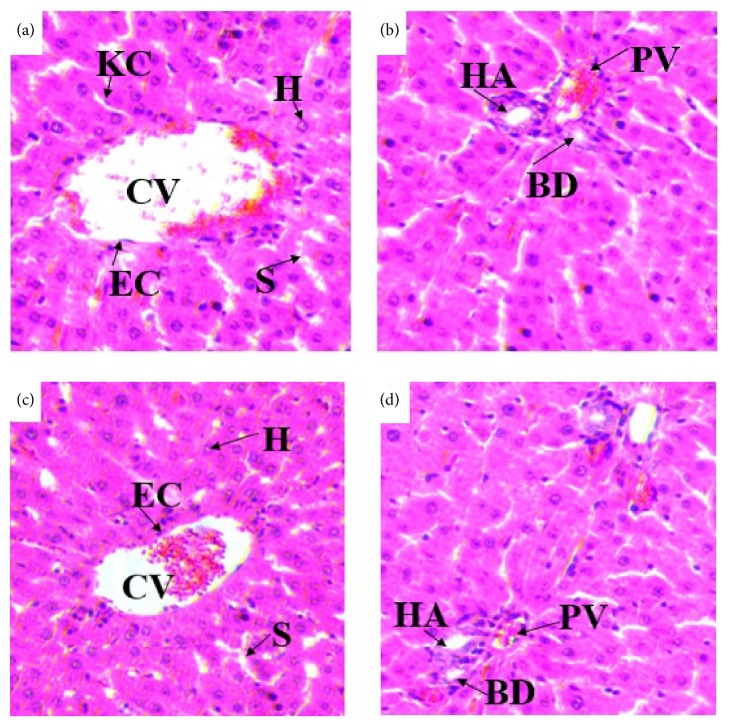
(a) and (b) Photomicrographs of liver section of rats treated with 1500mg/kg of 80% methanol extract of* S. guineense*. (c) and (d) Liver section of control rats (H and E, X400). CV= central vein, EC= endothelial cells, H= hepatocytes, KC= Kupffer cells, S= sinusoids, BD= bile duct, HA= hepatic artery, and PV= portal vein.

Histopathological studies of the liver sections in the control group (Figures 3(a) and 3(b)) showed normal appearance of central vein (CV) and hepatic sinusoids (S) lined by endothelial cells (EC) with normal radiating hepatocytes. There was also normal appearance of the portal triad including hepatic portal vein, interlobular bile duct, and branches of hepatic artery. Rats treated with 980% methanol extract of the leaves of* S. guineense* at both doses of 500 mg/kg (Figures 4(a) and 4(b)) and 1500 mg/kg (Figures 5(a) and 5(b)) showed normal appearance of the central veins (CV) and hepatic sinusoids (S) lined with endothelial cells (E) with normal radiating hepatocytes.

#### 3.2.5. Effects on Histology of the Kidneys

**Figure 6:**
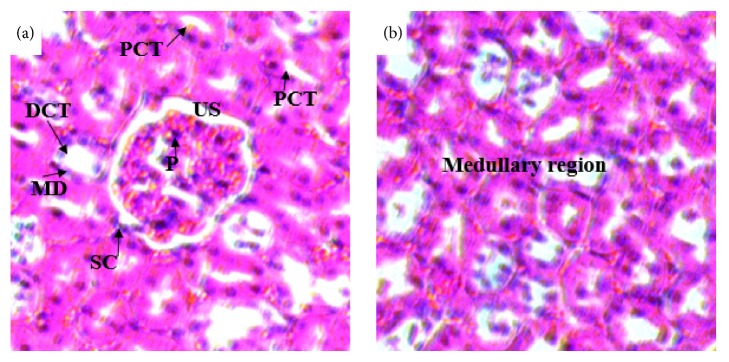
(a) and (b) Photomicrographs of the kidney sections of control rats (H & E x 400). PCT= proximal convoluted tubule, DCT= distal convoluted tubule, MD= macula densa, G= glomerulus, US= urinary space, SC= squamous cell, and P= podocyte.

**Figure 7:**
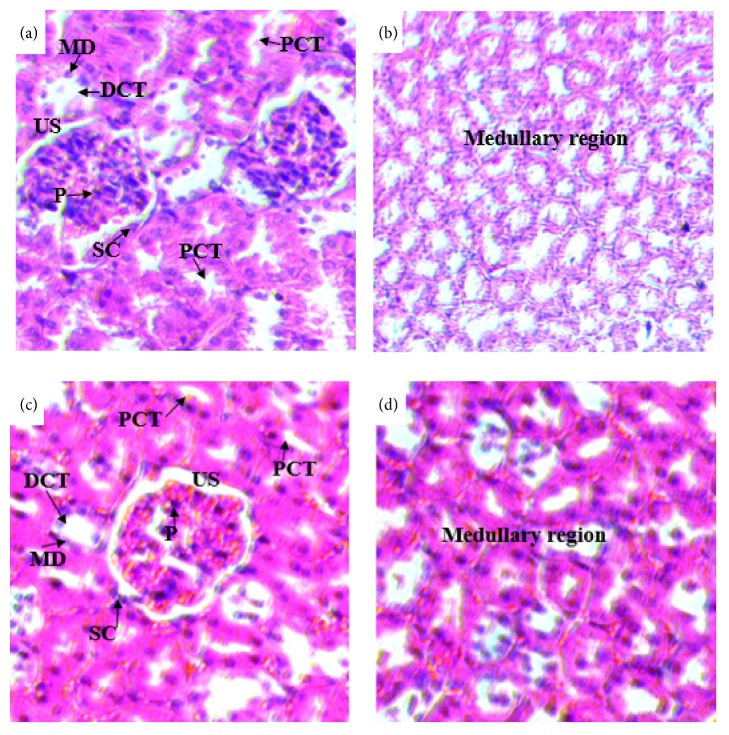
(a) and (b) Photomicrographs of the kidney sections of rats treated with 500mg/kg of 80% methanol extract of the leaves of* S. guineense* ((a) H&E-x400 and (b) H&E-x200) and (c) and (d) kidney sections of control rats (H&E-x400). PCT= proximal convoluted tubule, DCT= distal convoluted tubule, MD= macula densa, G= glomerulus, US= urinary space, SC= Squamous cell, and P= podocyte.

**Figure 8:**
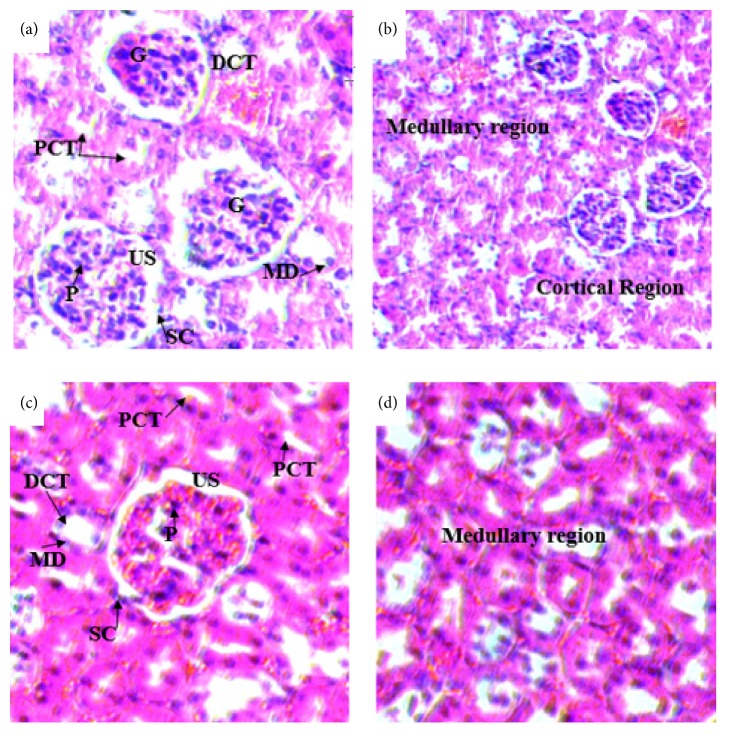
(a) and (b) Photomicrographs of the kidney sections of rats treated with 1500mg/kg ((a) H & E x400 and (b) H&E x200). (c) and (d) Kidney sections of control rats (H&E-x400). PCT= proximal convoluted tubule, DCT= distal convoluted tubule, MD= macula densa, G= glomerulus, US= urinary space, SC= squamous cell, and P= podocyte.

Histopathological studies of the kidneys sections of rats treated with doses of 500 mg/kg (Figures 7(a) and 7(b)) and 1500 mg/kg (Figures 8(c) and 8(d)) showed no significant microscopic changes compared with the controls (Figures 6(a) and 6(b)). But there were some changes in histology of kidney. In the treated rats of kidney sections revealed normal glomerulus (G), Bowman's capsule lined with outer parietal layer/squamous cells (SC) and inner visceral layer/podocytes (P), urinary space (US), proximal convoluted tubules (PCTs) lined by simple cuboidal epithelium with brush border, distal convoluted tubules (DCTs) lined by simple cuboidal epithelium with more nuclei per cross-section, and macula densa (MD) with taller cells around the vascular pole.

## 4. Discussion

### 4.1. Acute Toxicity Study

Acute toxicity test assesses the adverse effects that occur within a short timeafter administration of a single dose of a test substance. This testing is performed principally in rodents and is usually done early in the development of a new chemical or product to provide information on its potential toxicity [8].

For acute toxicity study, 80% methanol extract of* S. guineense *leaves were given to rats at a dose of 2000 mg/kg and 5000 mg/kg. The groups did not produce any signs of toxicity at both doses employed. No mortality was observed in the treated groups, i.e., at 2000 mg/kg and 5000 mg/kg during the study period. Therefore, the LD_50_ of the extract could be greater than 5000 mg/kg. The 80% methanol extract may, therefore, be considered relatively safe on acute exposure.

The present finding from the acute toxicity study agreed with the study reported by other researchers that there was nontoxic nature of the aqueous leaf extract of* S. guineense *up to 6000 mg/kg [18]. In addition, the study done on 80% methanol extract by Zeleke [17] showed that there was no sign of toxicity and mortality at 2000 mg/kg dose. Another study by Nigatu [32] showed that the LD_50_ (p.o.) of leaf tips aqueous extracts, twigs 80% methanolic extracts, stem bark aqueous extracts, stem bark 80% methanolic extracts, and fruit 80% methanolic extracts were 14100, 2910, 5120, 8770, and >10000 mg/kg, respectively. Another study done in Nigeria showed that the intraperitoneal LD_50_ of the ethanolic extract of* S. guineense* was found to be 3807 mg/kg [16].

Body weight change is an important index for assessment of toxicity [33]. In the present study, there was a gradual normal increase in the mean body weight of the treated groups like control group. The mean body weight gain for the control rats was 19.10 g. The mean body weight gain for rats treated with 2000 mg/kg and 5000 mg/kg was 18.9 g and 17.8 g, respectively. However, the weight gain difference between control and treatment groups was statistically insignificant.

Liver and kidneys of rats are used by many researchers to assess the safety or toxicity of drugs or plant materials [34]. In the current acute toxicity study, gross pathological examination of the liver and kidneys of treatment groups did not show any major visual difference in size, shape, color, and texture compared with control group. In addition, there was no significant difference in the absolute weight of liver and kidneys of treated rats compared to control group.

### 4.2. Subacute Toxicity Study

Subacute toxicity study examines toxicity caused by repeated dosing over an extended period of 28 days of oral administration in rodents. This test provides information on target organs and on the potential of the test chemical to accumulate in the organism and then is used as the basis for the determination of the no observed effect level (NOEL) [8].

In the present subacute toxicity study, the rats that were treated with 80% methanol extract of leaves at doses 500 mg/kg and 1500 mg/kg showed no signs of morbidity and mortality. During the experimental period no death or no apparent behavioral changes were observed compared with the control group. The current result disagreed with findings of study done on oral treatment of the aqueous leaf extract at dose of 200, 400, and 600mg/kg for six weeks in mice at which two mice died at days 32 and 40 from groups treated with 600 and 200mg/kg body weight of the extract, respectively [35]. The discrepancy between our findings with Amare's report might be because of the duration of exposure difference (42 days versus 28 days), species difference (mice versus rats), and the extracting solvent used (waters versus 80% methanol).

The gross pathological examination of the liver and kidneys of the treated rats showed no change in color, shape, size, and texture compared to the control group. According to Lu [36], remarkable change in relative organ weight between treated and untreated animals is an indicator of toxicity as organ weight is affected by the suppression of body weight. In the present study there was no significant change in weight of liver and kidneys of both treated groups compared to control group. This was inconsistent with the finding of Amare [35], who reported a significant increase in the liver weight ratio in mice treated with 200 mg/kg and a significant increase in the right kidney weight ratio in mice treated with 600 mg/kg.

During the subacute toxicity study, the treated rats showed gradual normal increase in their body weight. There was no significant difference in mean body weight gain of control, group I (500 mg/kg), and group II (1500 mg/kg) which were 58.52 g, 58.42 g, and 53.68 g, respectively. Increment in body weight determines the positive health status of the animals [37].

Assessment of hematological parameters can be used to determine the extent of harmful effect of foreign compounds including plant materials on blood [38]. In the present subacute toxicity study, the hematological parameters (RBC, WBC, PLT, HGB, HCT, MCV, MCH, and MCHC) were within the reference range for rats. The reference values of WBCs, RBCs, PLT, HGB, HCT, MCH, and MCHC for rats are 6–18 x 10^3^/*μ*L, 7–10 x 10^6^/*μ*L, 500–1,300 x 10^3^/*μ*L, 11–19.2 g/dl, 35–64%, 14.3–19.5 pg, and 26.2–40 g/dL, respectively [39, 40]. In the present study, the changes in hematological parameters of treated groups were not statistically significant compared with control group. This indicates that the extract may not possess toxic substance that can cause anemia or other abnormalities. This was in disagreement with the report by Amare [35] on some parameters stating a significant decrease in RBC and HGB count and significant increase in WBC count in treated mice compared to control group.

In toxicological evaluation, biochemical parameters have significant roles as a marker because of their response to clinical signs and symptoms produced by toxicants. Evaluation of hepatic and renal function is of prime importance to assess the toxic properties of extracts and drugs [41]. In the present study, all biochemical parameters did not show significant changes except mean values of serum glucose. The mean serum glucose level showed significant decrease at both administered doses compared with control. The result agreed with the study conducted on aqueous extract in normoglycemic and diabetic mice [18]. In contrast to this, the study conducted in Nigeria on antivenom studies of* S. guineense* extracts against* Naja katiensis* venom rats revealed that the methanol leaf extract has produced no significant change in blood glucose level [42].

Measurement of plasma urea has been used for many years as an indicator of kidney function. Plasma urea is usually increased in acute and chronic renal diseases. Urea clearance falls as the kidney fails and as a result, urea tends to accumulate with diseased kidneys that are unable to excrete these substances at normal rate; this will raise blood urea level [43, 44]. In normal adult rat serum is measured about 15-45 mg/dl [39]. In the present study, the mean values of urea have been shown with a slight increment at dose of 500 and 1500 mg/kg even though not significant and this was not associated with the histopathological changes of the kidney.

Creatinine is produced endogenously and released in to body fluids at a constant rate and its plasma concentration is maintained predominantly by glomerular filtration. Consequently, both plasma concentration and its renal clearance have been used as markers of the glomerular filtration rate [43]. In the current study, the mean amount of creatinine showed slight increment but not significant. The reference value of creatinine in adult rat is about 0.2–0.8 mg/dL [40]. In this study the measurement was within reference value and was supported by the absence of histopathological changes of the kidney.

Serum total protein change is caused by a change in the volume of plasma water and a change in the concentration of one or more specific proteins in the plasma. Decrease in the volume of plasma water (hyperproteinemia) is noted in cases of dehydration due to inadequate water intake or excessive water loss, as the case in severe vomiting or diarrhea [43]. The reference value of total protein in the serum of adult rat is in the range of 5.6–7.6 mg/dL [39]. In the current study, the amounts of total protein were slightly increased at dose of 1500 mg/kg compared to control but not statistically significant. The mean values of total protein were within the reference range for rats, which was also supported by the absence of histopathological changes in the kidneys of treated rats.

The abnormal elevation of the liver enzymes (ALT and AST) is usually associated with liver damage or alteration in bile flow. ALT is found primarily in the liver and is the most sensitive marker for liver cell damage. When a cell is damaged, it leaks this enzyme into the blood. AST is found primarily in the red blood cells, cardiac and skeletal muscles, and kidney. AST is not specific to liver as ALT. In this study, the mean values of AST and ALT at dose 500 mg/kg were high whereas at dose 1500mg/kg decreased compared with control, but it was not significant. This was supported by the absence of histopathological changes in the liver of treated rats.

The liver and kidneys have fundamental roles in the metabolism and excretion of drugs or plant products. Exogenous chemicals and their metabolites might result in toxicity or cell damage on these organs [45, 46]. In the current histopathological examination of the liver, rats treated with doses of 500 mg/kg and 1500 mg/kg of the 80% methanol extract of the leaves of* S. guineense* showed no change in the microscopic structure of the liver. The general architecture of the liver, appearance of the hepatocytes, the hepatic sinusoids, portal triads, and central veins are normal as compared with controls. The result was also accompanied by the nonadverse effects of the extract in any of the biochemical markers (such as ALT and AST), which showed statistically insignificant changes compared with control group [47]. This finding agreed with work of Amare [35] who reported that mice treated at a dose of 200 and 400 mg/kg body weight of the aqueous leaf extract of* S. guineense* showed no histopathological changes compared to control group, whereas the tissue morphology of mice treated with 600 mg/kg showed hemorrhagic centrilobular necrosis and fatty cytoplasmic vacuolation of the hepatocytes [35].

In the histopathological study of the kidney, rats treated with both doses (500 and 1500mg/kg) of the extract showed no significant difference compared to controls. The sections of the kidneys of treated rats showed normal general structure of the kidney and the normal appearance of glomeruli and tubules. The proximal convoluted tubules, distal convoluted tubules, and macula densa are intact. The result was further supported by the values of biochemical parameters of the blood (such as urea, creatinine, and total protein), which are main indicator of kidney damage [47]. This was in line with the work of Amare [35] who reported absence of difference in tissue morphology between control group and mice treated with the low dose, 200 mg/kg. On the contrary, the current finding disagreed with work of Amare [35] in which tissue morphology differences were observed in the kidney of mice treated with 400 and 600 mg/kg.

## 5. Conclusion

The acute toxicity study of the 80% methanol extract of the* S. guineense* leaves did not produce adverse effects on the behavior and gross pathology of the rats at treated doses. Therefore, the oral LD_50 _of the 80% methanol extract of the leaf of* S. guineense *was greater than 5000mg/kg. Meanwhile, subacute toxicity study of the 80% methanol extract of the* S. guineense* leaves did not adversely affect the body weight and hematological and biochemical parameters of tested doses. There were no signs of toxicity observed in the kidney and liver sections of treated rats. However, well designed subchronic and chronic toxicity studies should be carried out in order to set the clear picture of the safety of the plant part before developing* S. guineense* leaf based health product.

## Figures and Tables

**Table 1:** Effect of 80% methanol extract of *S. guineense* leaves on organ weights of rats during acute toxicity study.

Parameters	Doses	Mean	95% Confidence interval for mean		^*∗*^P value
			Lower Bound	Upper Bound	
Liver weight (g)	Controls	5.40	3.59	7.21	.248
	2000mg/kg	5.98	5.16	6.79	
	5000mg/kg	5.03	4.48	5.57	

Kidney weight (g)	Controls	1.15	.61	1.69	.932
	2000mg/kg	1.10	.92	1.28	
	5000mg/kg	1.15	.99	1.31	

^*∗*^One way ANOVA, n= 4/group.

**Table 2:** Comparison of the effect of 80% methanol extract of the leaves of *Syzygium guineense* on body weight of treated and control rats during acute toxicity study.

Parameters	Doses	Mean ± SEM	Significance
Body weight initial week 1	Control	5.63 ± 5.63	0.91
	2000 mg/kg	126.83 ± 3.14	
	5000 mg/kg	125.33 ± 3.04	

Body weight week 2	Control	134.90 ± 7.65	0.89
	2000 mg/kg	131.23 ± 3.08	
	5000 mg/kg	131.78 ± 5.68	

Body weight week 3	Control	146.93 ± 9.30	0.93
	2000 mg/kg	145.73 ± 5.49	
	5000 mg/kg	143.10 ± 6.80	

Values are expressed as mean ± SEM, N= 4/group.

**Table 3:** Effect of 80% methanol extract of the leaves of *S. guineense* on organ weight of treated and control rats during subacute toxicity study.

Parameters	Doses	Mean	95% Confidence Interval for Mean		^*∗*^P value
			Lower Bound	Upper Bound	
Liver weight (g)	7.03	6.02	8.05	7.03	.100
	7.70	6.63	8.77	7.70	
	6.40	5.43	7.37	6.40	

Kidney weight (g)	1.40	1.20	1.60	1.40	.082
	1.47	1.38	1.55	1.47	
	1.28	1.18	1.39	1.28	

^*∗*^One way ANOVA, n = 6/group.

**Table 4:** Effect of 80% methanol extract of leaves of *S. guineense* on hematological parameters.

Hematological parameters	Doses	Mean	95% Confidence interval for mean		^*∗*^P value
			Lower bound	Upper bound	
WBC x10^3^/*μ*L	Control	8.30	7.09	9.51	.384
	500mg/kg	8.13	7.23	9.03	
	1500mg/kg	9.45	6.67	12.23	

RBC x 10^6^/*μ*L	Control	7.31	6.92	7.70	.859
	500mg/kg	7.30	6.73	7.86	
	1500mg/kg	7.43	6.99	7.86	

HGB (g/dL)	Control	14.98	13.74	16.23	.693
	500mg/kg	15.35	14.75	15.95	
	1500mg/kg	15.40	14.50	16.30	

HCT (%)	Control	48.80	47.14	50.46	.814
	500mg/kg	49.18	45.90	52.47	
	1500mg/kg	49.80	46.55	53.05	

MCV (fL)	Control	66.80	64.26	69.34	.887
	500mg/kg	67.47	64.59	70.34	
	1500mg/kg	67.05	65.10	69.00	

MCH (pg)	Control	20.50	19.10	21.90	.618
	500mg/kg	21.12	19.99	22.25	
	1500mg/kg	20.75	19.97	21.53	

MCHC (g/dL)	Control	30.73	28.08	33.39	.894
	500mg/kg	31.30	29.50	33.10	
	1500mg/kg	30.98	29.02	32.95	

PLT x 10^3^/*μ*L	Control	697.17	603.01	791.32	.768
	500mg/kg	671.67	577.80	765.54	
	1500mg/kg	713.17	591.87	834.47	

^*∗*^One way ANOVA, n = 6/group.

**Table 5:** Effect of 80% methanol extract of leaves of *S. guineense* on biochemical parameters in subacute toxicity study.

Biochemical parameters	Dose	Mean	95% Confidence interval for mean		P value
			Lower Bound	Upper Bound	
AST (IU/L)	Controls	287.83	213.56	362.10	.181^*∗*^
	500mg/kg	325.83	296.77	354.90	
	1500mg/kg	257.50	181.82	333.18	

ALT (IU/L)	Controls	198.17	170.56	225.78	.257^*∗*^
	500mg/kg	228.50	181.83	275.17	
	1500mg/kg	193.67	150.80	236.54	

Urea (mg/dl)	Controls	51.33	46.25	56.42	.055^*∗*^
	500mg/kg	58.00	54.62	61.38	
	1500mg/kg	60.17	51.15	69.18	

Creatinine (mg/dl)	Controls	.72	.64	.80	.307^*∗*^
	500mg/kg	.78	.68	.89	
	1500mg/kg	.80	.69	.92	

Total Protein (mg/dl)	Controls	6.32	5.55	7.09	.891^*∗*^
	500mg/kg	6.28	5.73	6.84	
	1500mg/kg	6.43	6.04	6.83	

Glucose (mg/dl)	Controls	80.83	74.22	87.45	
	500mg/kg	53.83	49.72	57.95	.0001^*∗∗*^
	1500mg/kg	61.83	51.06	72.60	.013^*∗∗*^

^*∗*^ *One way ANOVA, n = 6/group.*

^*∗∗*^Post hoc analysis.

## Data Availability

The data used to support the findings of this study are included within the article.

## Abbreviations

AAU: Addis Ababa University
ALP: Alkaline phosphatase
ANOVA: Analysis of variance
ALT: Alanine transaminase
AST: Aspartate transaminase
BW: Body weight
DPX: Dibutyl phthalate in xylene
EDTA: Ethylene diamine tetra-acetic acid
EPHI: Ethiopian Public Health Institute
H & E: Haematoxylin and Eosin
HCT: Hematocrit
HGB: Haemoglobin
LD_50_: Median lethal dose
MCH: Mean corpuscular haemoglobin
MCHC: Mean corpuscular haemoglobin concentration
MCV: Mean corpuscular volume
OECD: Organization of Economic Cooperation and Development
PLT: Platelet count
RBC: Red blood cell
SPSS: Statistical package for social science
WBC: White blood cell
WHO: World Health Organization.
